# Supplementary material for: Towards water-free biobanks: long-term dry-preservation at room temperature of desiccation-sensitive enzyme luciferase in air-dried insect cells
Source: Sci Rep. 2017 Jul 26;7:6540. doi: 10.1038/s41598-017-06945-y (PMC5529557; doi:10.1038/s41598-017-06945-y)
Supplement: Supplementary file 1 — Supplementary Information [file 41598_2017_6945_MOESM1_ESM.pdf]

Towards water-free biobanks: long-term dry-preservation at room temperature of desiccation-sensitive enzyme luciferase in air-dried insect cells

Short title: Enzyme preservation at room temperature in air-dried insect cells

*Shingo Kikuta[1], Shunsuke J. Watanabe[1], Ryoichi Sato[1], Oleg Gusev[2,3,4], Alexander Nesmelov[4], Yoichiro Sogame[5], Richard Cornette[5], *Takahiro Kikawada[5,6]

[1]Graduate School of Bio-Applications and Systems Engineering, Tokyo University of Agriculture and Technology, Koganei, Tokyo 184-8588, Japan
[2]Division of Genomic Technologies, RIKEN Center for Life Science Technologies, Yokohama, Kanagawa 230-0045, Japan
[3]RIKEN Preventive Medicine & Diagnosis Innovation Program, RIKEN, Wako, Saitama 351-0198, Japan
[4]Institute of fundamental Medicine and Biology, Kazan Federal University, Kazan 420008, Russia
[5]Institute of Agrobiological Sciences, NARO, Tsukuba, Ibaraki 305-8634, Japan
[6]Department of Integrated Biosciences, Graduate School of Frontier Sciences, The University of Tokyo, Kashiwa, Chiba 277-8562, Japan

*Correspondences to
Shingo Kikuta
Graduate School of Bio-Applications and Systems Engineering (BASE), Tokyo University of Agriculture and Technology, Koganei, Tokyo 184-8588, Japan
Tel: +81-42-388-7277; Fax: +81-42-388-7277; E-mail address: singo@cc.tuat.ac.jp

Takahiro Kikawada
Institute of Agrobiological Sciences, NARO, Tsukuba, Ibaraki 305-8634, Japan
Tel: +81-29-838-6170; Fax: +81-29-838-6157; E-mail address: kikawada@affrc.go.jp

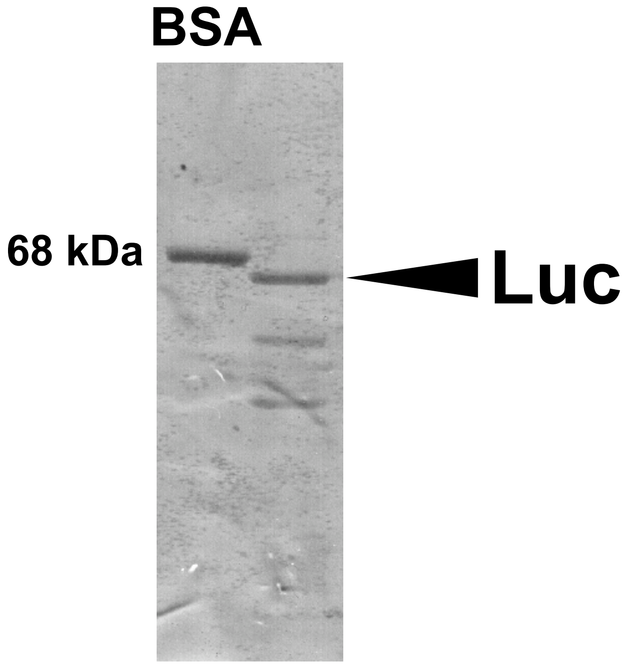

Figure S1
Recombinant luciferase obtained from *E. coli*. The samples were denatured at 95°C in SDS-PAGE sample buffer, the lysates were subjected to SDS-PAGE with 10% acrylamide gels, and separated proteins were stained with Coomassie brilliant blue. Arrowhead shows luciferase (Luc). BSA was used as a positive control.

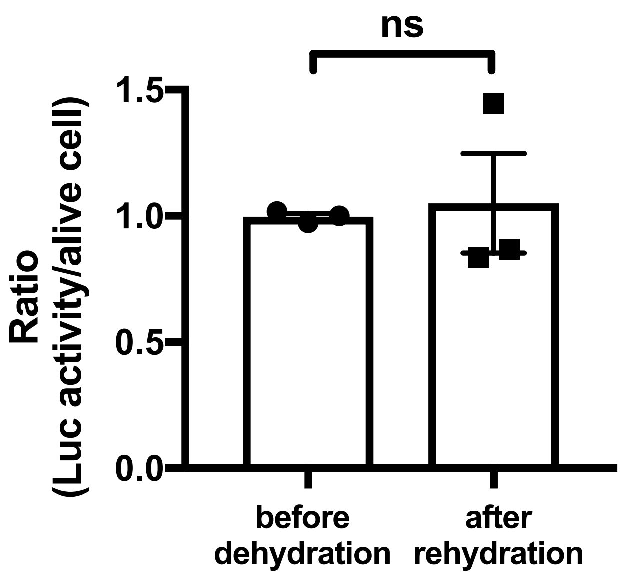

Figure S2

Luciferase activity per surviving cell by the ratio after rehydration. Survival of Pv11-Luc cells was examined using the calcein staining. Cell counting was performed after pre incubation with 600 mM trehalose and 1 h after rehydration. Each value is shown as a scatter dot plot. Error bar shows standard error of mean (n = 3). Statistical significance was evaluated with Mann-Whitney *U* test (p = 0.35).

A

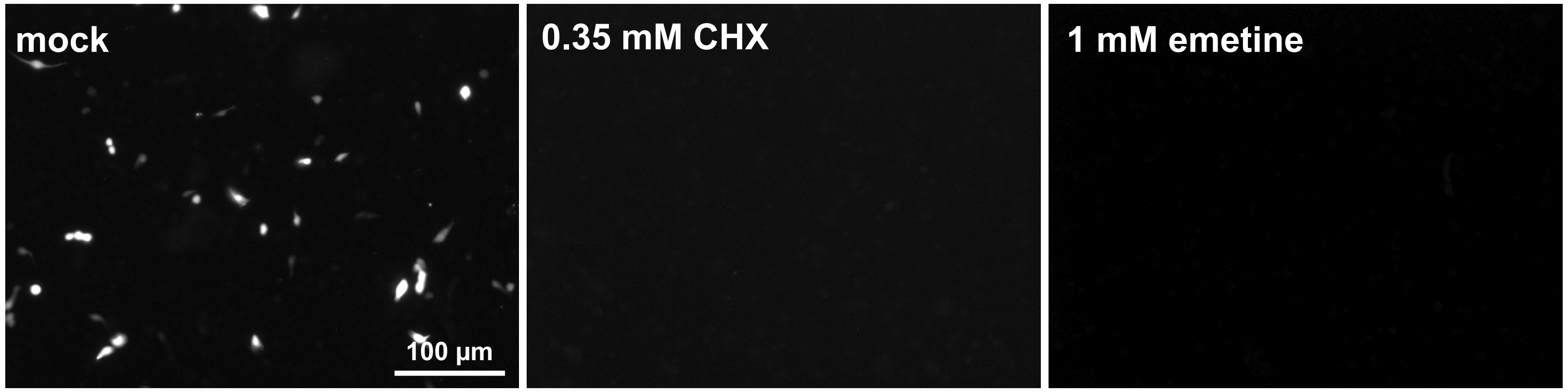

B

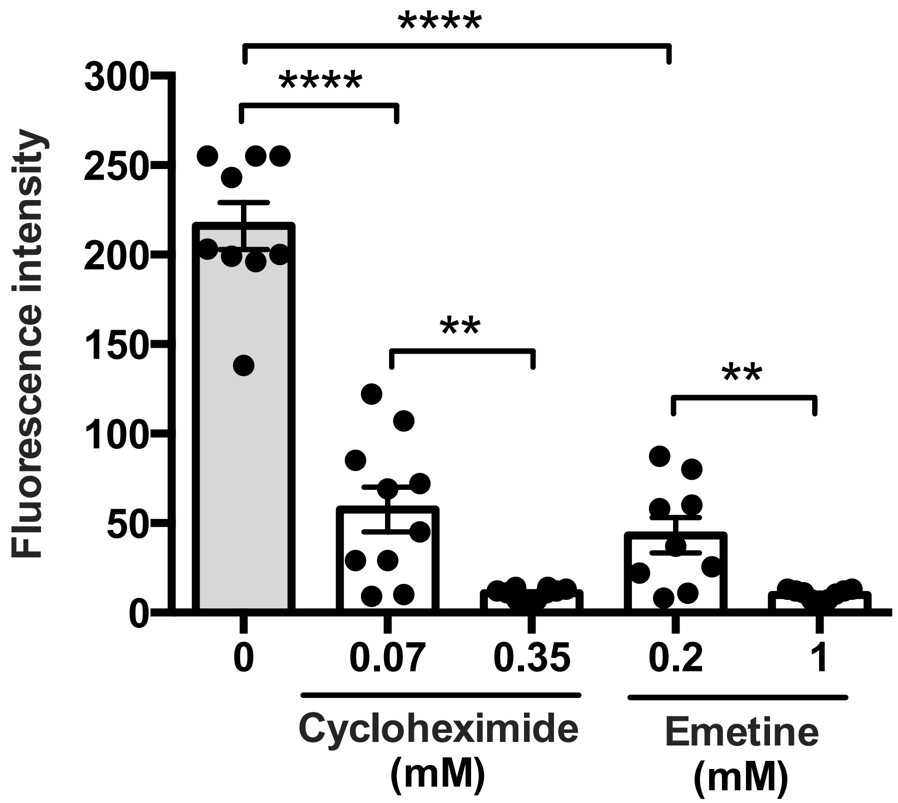

Figure S3

The effective concentration range of translation inhibitors for Pv11 cells was determined using the transient gene expression system. AcGFP1 fluorescence was observed at 48 h after transfection into Pv11 cells. Each translation inhibitor in different concentrations was added into the culture medium after transfection to Pv11 cells. **A**. AcGFP1 fluorescence imaging. Mock shows the AcGFP1 fluorescence without inhibitor. **B**. Fluorescence intensity of AcGFP1 in randomly picked-up individual cells was measured 48 h after transfection, and is shown as a scatter dot plot. Error bar shows standard error of mean (n = 9–10). Statistical significance was calculated by using one-way ANOVA and Tukey's multiple comparison test as a post hoc analysis ("****" means significantly different $p < 0.0001$; "**" means $p = 0.002$ for cycloheximide; $p = 0.004$ for emetine; "ns" means not significantly different).
